# Toward a new paradigm of DNA writing using a massively parallel sequencing platform and degenerate oligonucleotide

**DOI:** 10.1038/srep37176

**Published:** 2016-11-23

**Authors:** Byungjin Hwang, Duhee Bang

**Affiliations:** 1 Department of Chemistry, Yonsei University, Seoul 120-749, Republic of Korea

## Abstract

All synthetic DNA materials require prior programming of the building blocks of the oligonucleotide sequences. The development of a programmable microarray platform provides cost-effective and time-efficient solutions in the field of data storage using DNA. However, the scalability of the synthesis is not on par with the accelerating sequencing capacity. Here, we report on a new paradigm of generating genetic material (writing) using a degenerate oligonucleotide and optomechanical retrieval method that leverages sequencing (reading) throughput to generate the desired number of oligonucleotides. As a proof of concept, we demonstrate the feasibility of our concept in digital information storage in DNA. In simulation, the ability to store data is expected to exponentially increase with increase in degenerate space. The present study highlights the major framework change in conventional DNA writing paradigm as a sequencer itself can become a potential source of making genetic materials.

The surge of big data in various disciplines poses challenges in digital preservation. Most recording media, including magnetic and optics, are vulnerable to deterioration and, even worse, are associated with the problem of obsolescence in retrieval and playback technologies because related industries are growing at a rapid pace. Recently, several groups of researchers conducted experiments using DNA as a digital archive due to its stable and ultra-dense nature^1^,^2^. Although recent advances in programmable microarray synthesis have expanded the throughput up to the millions of oligonucleotides per chip, performing a large number of testing hypotheses on a given array is still daunting task. Furthermore, although it is difficult to predict in a precise manner, fabrication of DNA microarray into sub-micron scale is challenging as an elaborate control of simultaneous deposition of DNA bases is required for constructing microarray chip. Unlike the DNA writing field, the sequencing cost per base has been dropping precipitously in the last few years owing much to innovative technologies and competition among industries. Thus, a widening of the throughput capacity gap between the state-of-the art DNA writing and reading is inevitable, which will eventually limit the scalable use of synthetic DNA for making high capacity genetic or memory materials.

## Results

### Library construction using degenerate oligonucleotide

**Figure 1:**
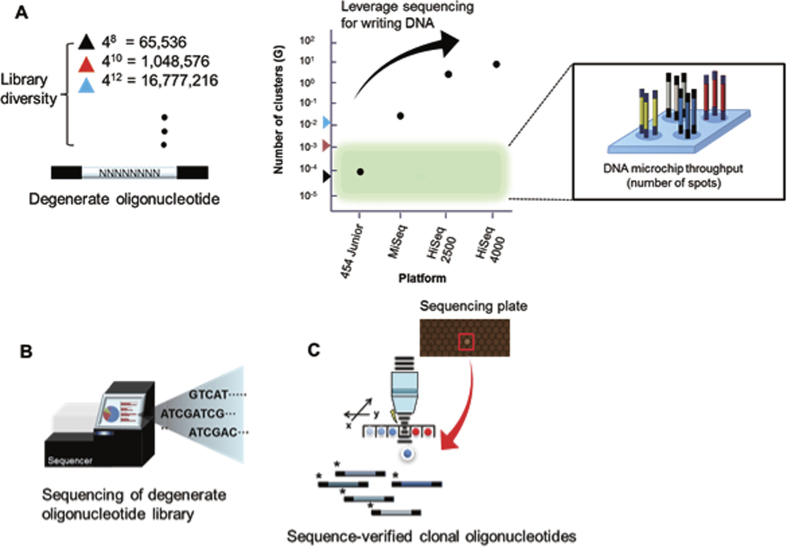
Leverage sequencing capacity for synthesizing raw genetic materials. Accelerating sequencing throughput by contrast to technologies related to writing DNA. (**A**) Degenerate oligonucleotide provides diverse clonal library (N can be A, T, C or G). The current high-density microarray platform covers around a million scale oligonucleotides in one chip (shaded region represents the range of number of spots in current microarray platforms). Colored triangle in library diversity corresponds to the throughput in terms of number of clusters (reads). (**B**) Sequencing of degenerate oligonucleotides in the sequencing plate provides a rich source of DNA materials. The sequencing throughput far exceeds the current state-of-the art writing technologies and may become a potential source of making standard genetic materials. (**C**) After sequencing, mapping the physical location of the clonal sequence information with actual pixel image enables the retrieval of sequence-verified clonal information that will be used as subsequent building blocks for making genetic materials.

To address the gap, we present a method for a scalable generation of desired DNA molecules using only one oligonucleotide and next-generation sequencing (NGS), without the need for a microarray synthesis. We hypothesize that the sequencing plate itself can be a rich source of clonal sequences that circumvents the need for a multiple array synthesis (Fig. 1. The throughput in terms of number of clusters in sequencing corresponds to the number of spots on the array).

**Figure 2:**
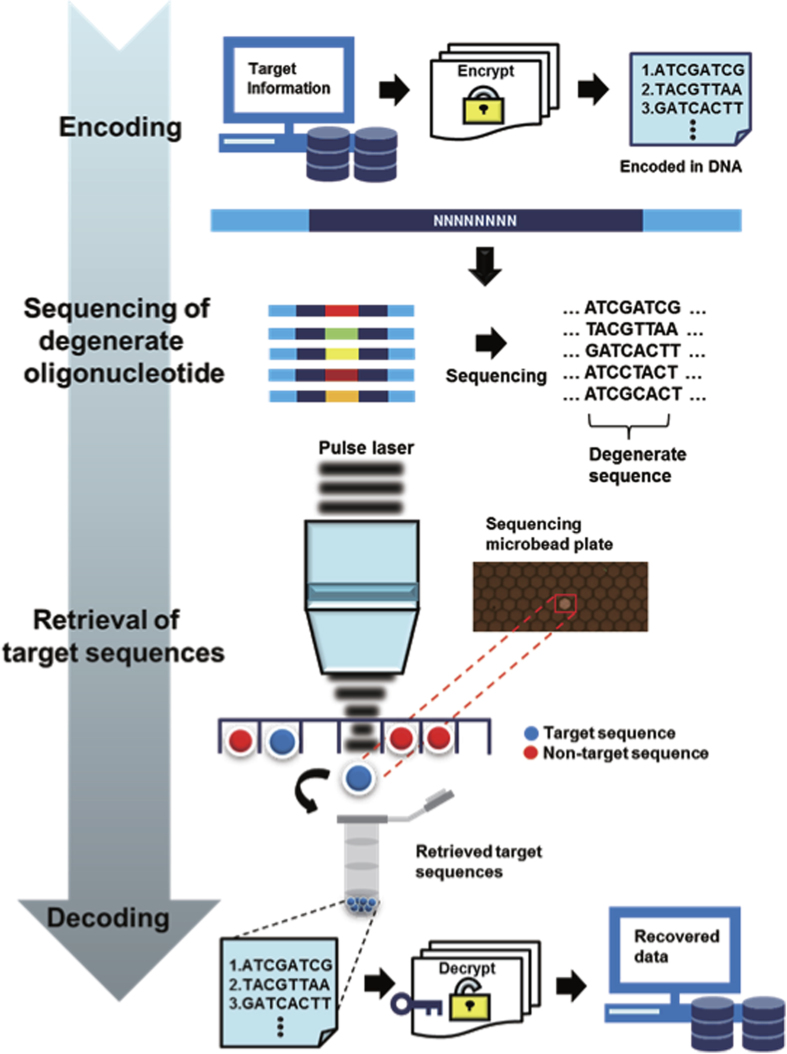
Schematic flow of high-throughput data storage in DNA. From a single degenerate oligonucleotide, diverse sequence information can be extracted using high-throughput sequencing (reading DNA). Information is encoded with the Huffman code in DNA using the base-4 scheme. The sequencing of a degenerate oligonucleotide generates diverse clonal sequences. Clonal sequences are then retrieved in a tube using an automated laser pulse. Additional tubes can be used to separately collect duplicate information for correcting errors (see Fig. 3). The pool consisting of the targeted sequence is sequenced to recover the original information. The decoding process is exactly the reverse of the encoding process. After sequencing, an *in silico* assembly is performed to recover the original data.

The proposed DNA writing was composed of the following steps (Fig. 2); synthesis of a short oligonucleotide with degenerate nucleotides, running NGS of the oligonucleotide to generate maximum possible clonal DNA materials, mapping sequence information along with positional coordinates on the surface of the NGS flow-cell, and subsequent target DNA retrieval from the flow cell onto a collection tube. Finally, the DNA retrieved from the NGS plate can be decoded via the NGS platform. In this experimental setting, the capacity of DNA synthesis can be equalized with the sequencing capacity.

To demonstrate the validity of the proposed method, we conducted a proof of principle experiment for the construction of DNA storage using only one synthetic oligonucleotide containing degenerate random eight ‘N’ (A, T, C, or G) sequences (up to 4^8^ diversities) flanked by NGS adaptor sequences. To prepare NGS libraries, we converted single-stranded oligonucleotides to double-stranded oligonucleotides by annealing and extending one reverse primer. We then applied the Roche 454 Junior system to the sequence library containing random degenerate sequences and obtained these clonal sequences (Figures S1 and S2).

### Encoding and decoding DNA data storage

**Figure 3:**
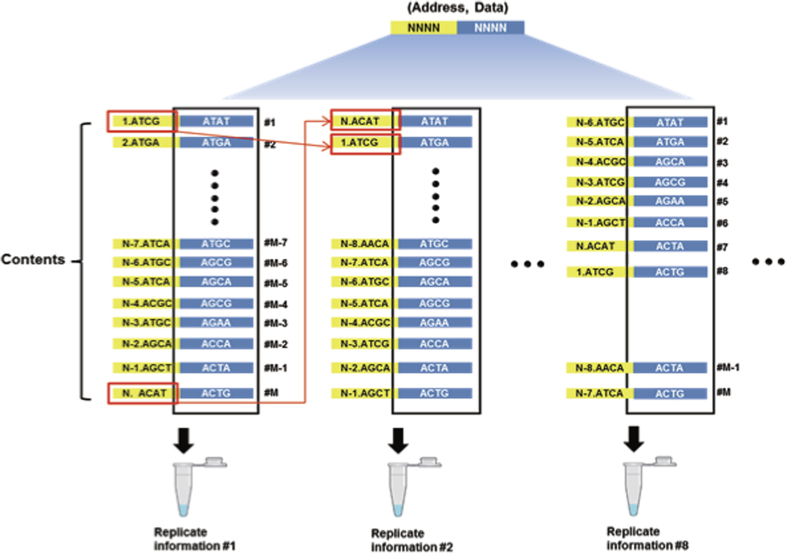
Tiling strategy. In our study, the sequence space is bounded by 8-mer due to the throughput of the 454 Junior sequencer. Thus, multi-depth tiling is crucial as error in the address or data part could prohibit a perfect assembly of the original texts. We expected that this would be resolved as degenerate nucleotide space increases. The numbers in the yellow (address) block represents the data location of the address sequences (denoted by 1…N). One address sequence slides consecutively for each tiling step as indicated by the red arrow. Sequences corresponding to the same row (black box) are the same for each data block (denoted by 1…M). Illumina adaptors are ligated for each tube to make final sequencing library for decoding of original information (see Figure S1).

To show arbitrary DNA-based storage, we first used the memory-efficient Huffman coding strategy^3^ to convert selected text (377 words) from a representative paper^4^ to base-4 digits, giving a total Shannon information^3^ of 1569 bits (see Methods for details). The scheme was based on a variable-length table where fewer bits represent more common characters. The bit information from each file was encoded into a 4-bit data location block (4 nt) and 4-bit data block (4 nt). Given that bases with synthesis or sequencing errors exist and the synthesis process is stochastic, we designed an 8-fold coverage by sliding the address block DNA sequences. The address bases are shifted to subsequent address block for making redundant information (Fig. 3). This approach allowed us to utilize the remainder of the sequenced library. Last, these strings consisted of (0, 1, 2, and 3) were converted to single DNA bases as described before with modification^2^. Following the sequencing of the degenerate oligonucleotide library, a high-throughput optomechanical system based on non-contact laser pulse^5^ was used to retrieve a target sequence containing beads from the 454 sequencing plate. Briefly, 454 sequencing plate (chip) is first located in the optical retrieval system. The beads are extracted using direct ablation of the laser pulse and re-amplified and evaluated for Sanger sequencing for initial mapping step. Then, mapping of the pixel information of the sequence to physical location of the beads is performed (x, y read locations registered in the sequencing output). Aided by a linearized motor stage, a radiation system allowed us to separate beads automatically. To read and reconstruct the text, beads collected in tubes were amplified and sequenced on an Illumina HiSeq platform (Figure S3). Low-quality raw reads were trimmed and expected oligonucleotide lengths were examined. For the Illumina data, we joined overlapping reads from 150 bp paired-end reads to filter sequencing errors. After filtering perfect index or address sequences (mostly substitution errors), we generated consensus bases using the majority selection rule. The original text was recovered with 100% accuracy, determined by examining the consensus sequences (see Methods for details).

### Data encoding capability

**Figure 4:**
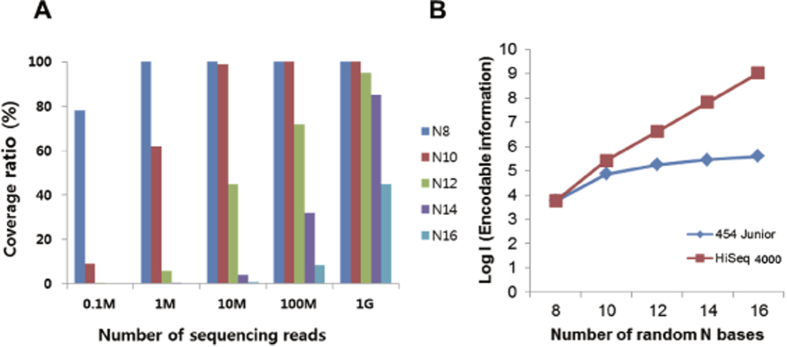
Simulation of degenerate oligonucleotide space. (**A**) A simulation was conducted to estimate the coverage ratio of the corresponding degenerate oligonucleotides (N8 refers to the oligonucleotide with eight random octamers). Given the same sequencing capacity, the coverage ratio was reduced as the diversity of the oligonucleotide increased (1 G refers to a billion reads and the error bars represent mean + s.e.m from 10 Monte Carlo simulations). (**B**) Simulation of data encoding capability. We calculated the theoretical maximum amount of encoding information (454 Junior reads up to 150 k and 2 billion for HiSeq 4000). The equation for the above graph was derived from a calculation similar to Goldman *et al*. (*2*). Let B be the number of bases for the address block and A be a number of bases for the data block and C is coverage. Then, A + B = N (1). Intuitively, the optimal relationship for B and C is B = Ceil[log_4_(C)] for base-4 (converted from bytes using the Huffman code). The relationship for the number of required DNA base (b) and coverage is b = C × A. It is clear that the expression can be rewritten as b = C × [N − Ceil(log_4_C)]. For our experimental setting, b = 2.85 × I (2.85 DNA bases per byte in Shannon information).

Next, we simulated the effect of the degenerate space on the coverage of the DNA species. Distinctive DNA sequences with uniform probability were generated, from which cumulative counts were enumerated for the subsampled sequencing reads. As the size of the degenerate space increased, the coverage ratio decreased in a given sequencing capacity (Fig. 4A). Specifically, the greater the sequencing capacity, the lower the coefficient of variation (Figure S4). Assuming the coverage ratio (y) and the sequencing reads (x) are a monotonically increasing function, the coverage ratio increases as more nucleotides are sequenced. Our application could include two kinds of bias. For the synthesis bias, some sequences could appear more than others. Based on Figure S5, the unequal distribution of contents exhibited a slightly lower coverage ratio than the uniform one. In addition, the target information to be stored could have a sequence distribution bias. Next, we simulated large-scale scenarios by extending the current 454 based DNA writing platform to a more high-throughput sequencing platform. Assuming all synthetic combinations were present in the degenerate library, we also calculated the number of encodable information as the number of degenerate space increased. As degenerate N bases increase up to 16 bases (at least 4^16^ ≈ 4*10^9^ reads required), the maximal encodable information increases exponentially (Fig. 4B). However, in reality, a base composition bias may exist in the oligonucleotide synthesis step. We performed saturation analyses using the down-sampling method and estimated that the number of available barcodes was approaching saturation (Figure S6). If degenerate space increases, this scenario is not compatible with the performance of the current system (454 Junior, ~100,000 reads). Thus, an upgrade of the current optomechanical DNA retrieval system to Illumina’s HiSeq platform would eventually cover all possible combinations (4^N^; N: number of degenerate bases). We have shown the potential for target sequence retrieval based on the MiSeq platform in a previous study^5^.

## Discussion

In our experiment, errors could occur during the synthesis step and the sequencing capacity of a 454 system would hamper reconstruction of the original data. To address this, tiled sequences (encoding the same information) were placed in separate tubes because of limitation in the address block size (4^4^ = 256). On average, 92% of the clonal oligonucleotides required for constructing the entire text in each tube were obtained. Missing sequence information was supplemented from the other independent tubes (multiple copies of the clonal sequences encoding for the same content were present). Moreover, PCR amplification of the pooled clonal sequences in the tube and the sequencing step may affect the overall performance. However, we found that our mean sequencing coverage over the encoded location was sufficient for decoding the contents.

Following points are notable for discussion. First, our platform poses both a synthesis bias and a target information bias. To address both issues, different encoding schemes can be used. For example, Church *et al*.^1^ allowed alternative DNA sequences to be encoded for the same contents (A or C for 0 and G or T for 1). Incorporating a parity strand based on the XOR operation^6^ might extend the possibility to encode information using different oligonucleotide sequences. Further, the sliding approach could be used to resolve these issues. Although a simple sliding frame was utilized, a more complex rule-based method would extend the maximal usage of the address block combinations. By virtue of the sliding approach, we could overcome the limitation of degenerate space. In this case, separate tubes were incorporated to make a replicate copy of the encoded text to compensate for the loss in other tubes. If the throughput of the sequencer increased, then multiple copies of the same contents could be obtained. Indeed, sequences from both data and the address part would become unique as random ‘N’ sequence space increased. Hence, the probability of finding the same particular sequence among different tubes would also be minimized. Second, error seems negligible in recovering the original information with increasing data size and the only limiting factor was in the sequencing output. Third, a more elaborate error-correction coding scheme could be further integrated for efficient information storage. Finally, to target a specific sequence in a high-throughput manner, a fast and high-resolution laser system could be used to detach DNA molecules from the flowcell^7^,^8^. In this way, a significant advance in the throughput can be achieved compared with the robotic pick-in-place oligo retrieval methodology^9^.

Until now, the scalability of DNA as data storage was limited by microarray synthesis density. However, our method depends on the accuracy and the rate of retrieval of the target DNA. This fact could have important implication for ‘DNA writing’ technology, especially in the field of gene synthesis. Considering that the major breakthrough in artificial gene synthesis was first demonstrated^10^ with short oligonucleotides (~20 mer), we envision that a new paradigm for a massively parallel synthesis of genetic materials can be realized in the future with NGS sequencer *per se* as a source of building blocks of oligonucleotides. However, several technical challenges regarding this issue remain. First, as shown in Fig. 4B, the number of obtainable DNA species is limited by the throughput of contemporary sequencing technologies. Second, GC rich sequences or homopolymers^11^ would be difficult to retrieve if a synthesis bias occurs. Using genetic code redundancy could partially resolve this issue. In addition, iterative enzyme digestion and the ligation of short dsDNAs could be utilized for full-length, gene-sized assembly^12^.

Despite these limitations, we addressed the key challenge of making genetic material leveraging sequencing capacity in DNA storage application. Initially, we made use of a single degenerate oligonucleotide to harness a vast amount of sequence information. Sequencing was then used to profile this rich source of information. Desired clonal sequences could be accurately retrieved with precise targeting by automated pulse laser. Coupled with the fast and accurate high-throughput retrieval method, our method could be easily scaled up to cater for current massively parallel sequencing technologies. Although we relied on one framework in our evidence-of-concept, it is reasonable to expect that the amount of encodable information would exponentially increase with the aid of high-throughput NGS system.

## Methods

### Encoding of digital information

The representative English text (part of the abstract, given *Italic* below) was selected from a key paper in genomics as referred to in the main text. From this text, 377 characters (bytes) were converted to Shannon information of 1569 bits using the Huffman encoding strategy.

“*The human genome holds an extraordinary trove of information about human development, physiology, medicine and evolution. Here we report the results of an international collaboration to produce and make freely available a draft sequence of the human genome. We also present an initial analysis of the data, describing some of the insights that can be gleaned from the sequence*.”

Each character was encoded as a sequences of DNA bases using base-4 digits (0, 1, 2 and 3 for T, C, G and A). If the first character starts with “T” (0) and subsequent code is 2, then next nucleotide would be “A” (Figure S7).

For example, using the first 3 words from the above text, “the human genome” can be first converted using Huffman tree.


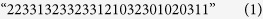


Since location index and data encoding part is 4 bp each, add ‘0’s if not divisible by 4. From (1), add one ‘0’ and split code by 7 groups (length = 28, 28/4 = 7).


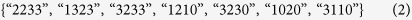


Then, add location index code {‘0000’, ‘0001’, ‘0002’, ‘0003’, ‘0010’, ‘0011’, ‘0012’} ahead of the each code in (2) which yields:


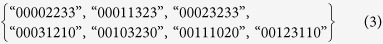


These codes were converted to DNA sequences using the strategy (Figure S7) above. The 8-mer containing oligonucleotides were retrieved using high-throughput laser system in the subsequent step. The source code for data encoding and decoding process is written in python and available in Supplementary Software (demo. py).

Our data encoding scheme achieves 2.85 DNA bases per byte in Shannon information, whereas the theoretical maximum of encodable DNA bases in a base-4 encoding system per byte is log (27)/log (4) = 2.38 (for 27 symbols used in our study). To achieve an optimal compressed system, each byte (symbol) should be equally frequently appeared and be purposely designed. Here, however, we have selected a document for a conceptual presentation.

### NGS (454 Junior) Library construction and sequencing

A single-primer extension was conducted using 1 μl (10 μM) of 454 reverse primer (5′-CCTATCCCCTGTGTGCCTTGGCAGTCTCAG-3′) with 1 μl (10 μM) of template oligonucleotide and 8 μl of D.W. and 10 μl of KAPA HiFi HotStart Ready Mix (2×) to reduce PCR bias using the following cycle conditions (10 μM primer): denaturation at 98 °C for 3 min; ramp down to annealing temperature (60 °C) at 0.1 °C/sec; and final extension for 5 min at 72 °C. The DNA sample quality was assessed by a Qubit 2.0 fluorometer. A quantified DNA library was sequenced with the 454 Junior platform (read length, ~400 bp). We obtained a total of 11,394,840 bp. After aligning to the reference using Bowtie2 version 2.2.4 (97.6% average alignment with the –end-to-end mode), we filtered reads with an expected length (138 bp, 88.5% on average) and further inspected the distribution of random octamer sequences (Figure S2).

Individual clonal sequences were extracted from the 454 sequencing plate using Sniper cloning and the pooled oligonucleotides were amplified using primers (eight combinations of forward and reverse NEBNext Mutiplex Oligos for Illumina) including specific index sequences for tiling information (Figure S1).

### Recovery of original templates using high-throughput sequencing platforms

As shown in Fig. 3, each replicate (encoding for the same text) tiled contents should be placed in the separate tubes. For each tube (the maximum number of addresses are 4^4^ = 256), tube specific index sequences are used to amplify the pool of retrieved target clones. PCR conditions were as follows: 25 μl of KAPA HiFi HotStart Ready Mix (2×) and 1 μl (10 μM) of each primer with cycling conditions of initial denaturation at 98 °C for 3 min, followed by 20 cycles at 98 °C for 30 s, 60 °C for 30 s, 72 °C for 1 min, and a final elongation at 72 °C for 5 min. For decoding (validation) process, different index sequences should be used to distinguish the contents of the different tubes as they are pooled for a final sequencing step. For example, in Fig. 3, the N-th address information (i.e., ACAT) is also found in a second tube. Therefore, the ordering of the sequence information for the decoding process is crucial. Finally, we could leverage Illumina’s high-capacity for sequencing output while maintaining high quality to decode original information. Trimmomatic^13^ was used to trim low-quality reads from paired-end Illumina sequencing. Duplicate information (reads) can be obtained and a majority voting scheme was implemented to correctly assemble the entire context *in silico*.

## Additional Information

**How to cite this article**: Hwang, B. and Bang, D. Toward a new paradigm of DNA writing using a massively parallel sequencing platform and degenerate oligonucleotide. *Sci. Rep.* **6**, 37176; doi: 10.1038/srep37176 (2016).

**Publisher's note:** Springer Nature remains neutral with regard to jurisdictional claims in published maps and institutional affiliations.

## Supplementary Material

Supplementary Information
