# Supplementary material for: Toward a new paradigm of DNA writing using a massively parallel sequencing platform and degenerate oligonucleotide
Source: Sci Rep. 2016 Nov 23;6:37176. doi: 10.1038/srep37176 (PMC5120280; doi:10.1038/srep37176)
Supplement: Supplementary Information [file srep37176-s1.pdf]

Supporting Information

Toward a new paradigm of DNA writing using a massively parallel sequencing platform and degenerate oligonucleotide

Byungjin Hwang, Duhee Bang*

Department of Chemistry, Yonsei University, Seoul 120-749, Republic of Korea

*Corresponding author: duheebang@yonsei.ac.kr

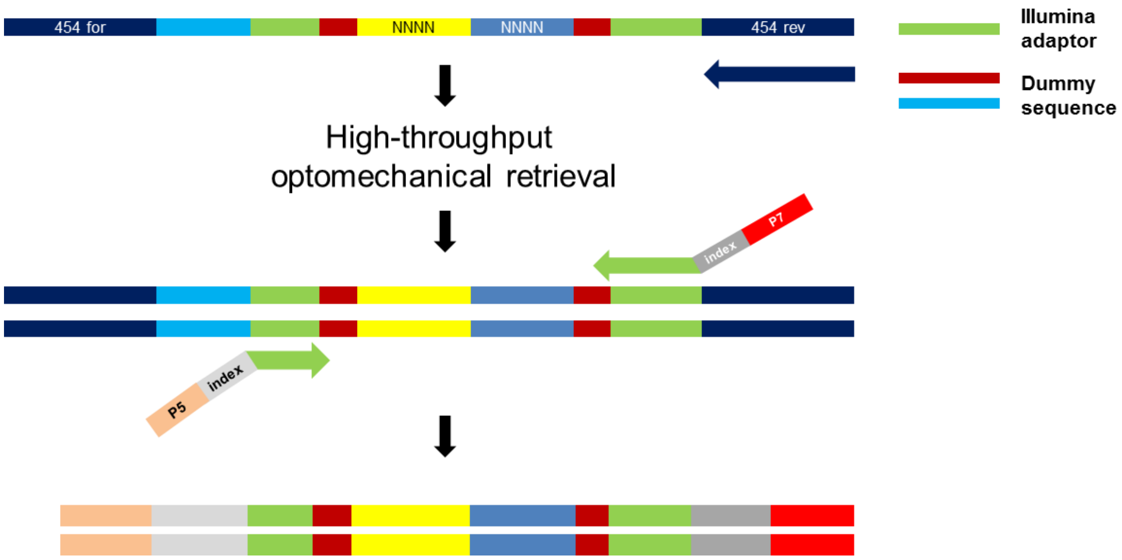

Figure S1. Oligonucleotide design and library construction. We designed oligonucleotides containing a location (4 nt) and data (4 nt) block. We limited the space of degenerate nucleotides to 8-mers because the 454 Junior system's capacity is ~100,000 reads (the maximum diversity of our design is 4^8). At the end of both oligonucleotides, 30 bp adaptor sequences for 454 Junior sequencing were attached; our optomechanical retrieval system was developed based on 454 sequencing plates. Random DNA sequences (8bp, underlined) were added to maximize the sequencing yields as empirical evidence in our lab has shown that amplicon sequencing in the 454 Junior had a common problem of signal cross-talk interference leading to low yields. An additional dummy sequence was added for compatibility with a high-throughput sequencing system (for stable cluster generation in sequencing) considering the following properties: no homopolymer stretches >4 bp, GC % between 40%-60%. Illumina adaptor sequences were also added to handle sequence diversity as degenerate space increased. Ultramer oligonucleotides were purchased from Integrated DNA Technologies (IDT) with a standard desalting technique. Sequences are given below.

('CCATCTCATCCCTGCGTGTCTCCGACTCAGNNNNNNNNACACTCTTTCCCTACACGACGCTCTTCCGATCTGATGCCTATGACCTGAGATGTTAGATGANNNNNNNNTTCCTGGTGTTACAGCTTCACTAGGAGAGATCGGAAGAGCACACGTCTGAACTCCAGTCACCTGAGACTGCCAAGGCACACAGGGGATAGG').

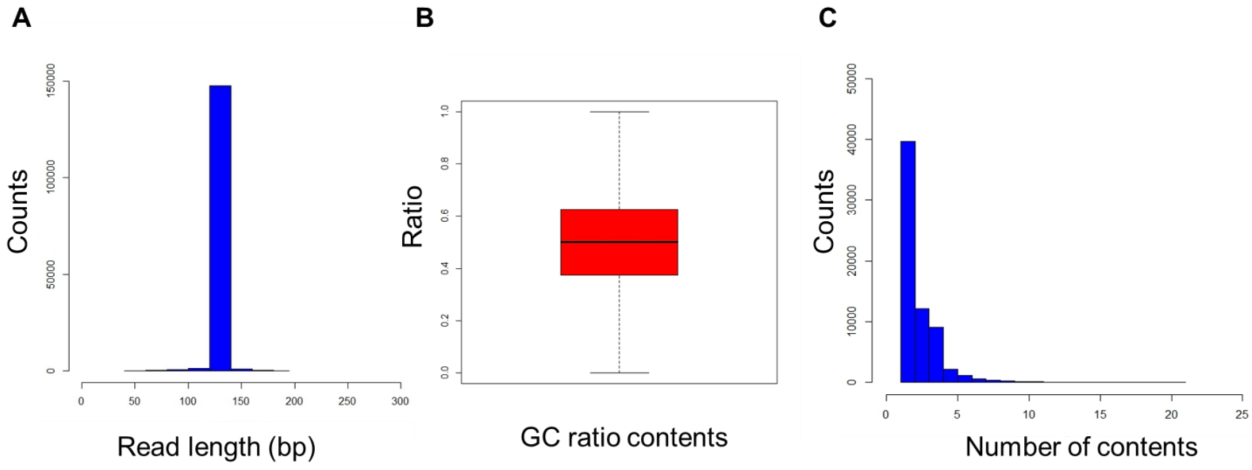

Figure S2. Distribution statistics after sequencing of the initial degenerate oligonucleotide. (A) Read length distribution of the initially sequenced oligonucleotide library. (B) GC ratio of the sequenced library (average: 0.49). (C) The majority of the degenerate octamer contents appeared < 3 times (>=80%) and few were overrepresented (>=20).

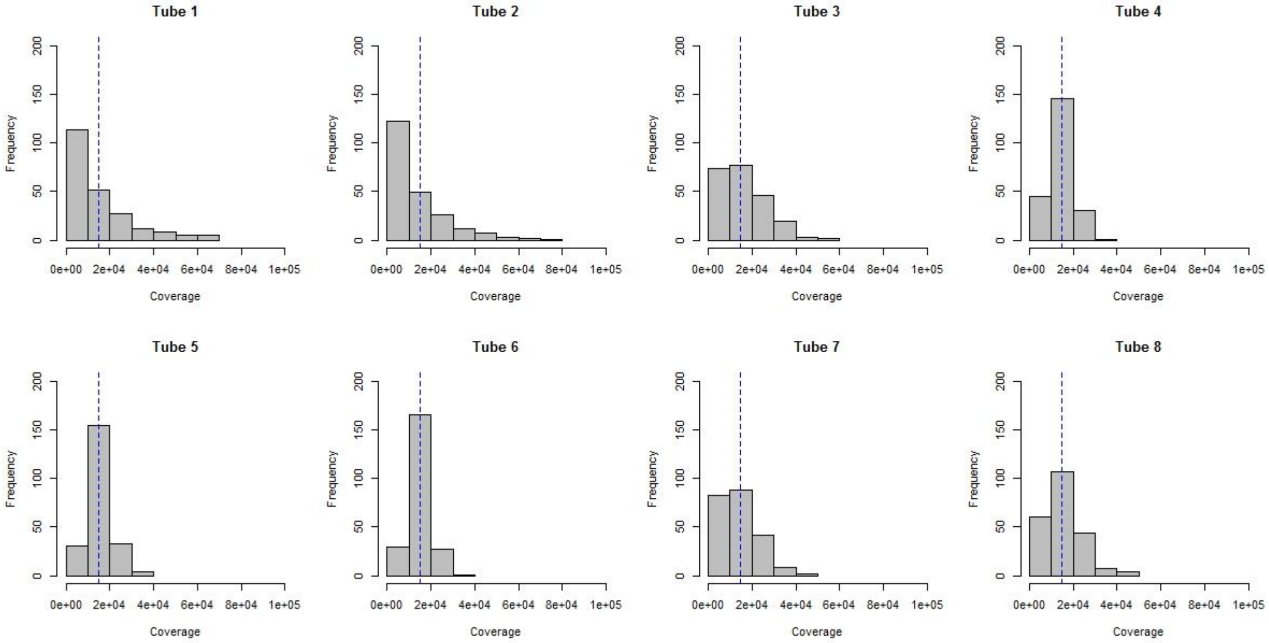

Figure S3. Coverage distribution of eight replicate tubes from encoded text. Amplification bias over the entire encoding location results in a large variance within the individual tubes but a small variation among the tubes, which enabled reconstruction of the whole text. The dotted blue line refers to the mean value of the read depth. Pooled data from eight tubes were sequenced using 8% of the HiSeq4000 1 lane (1% for each tube). The X-axis refers to the coverage of each of the contents over the encoding location and the Y-axis refers to the count of each coverage.

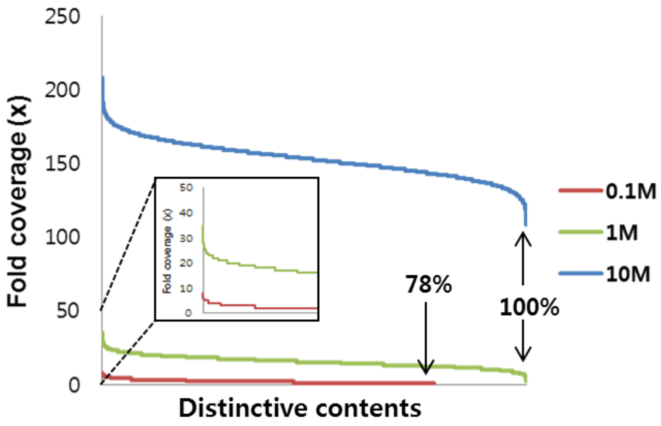

Figure S4. A simulation of the encoding space in the current study. The enumeration of distinctive DNA sequences from the uniform probability for an oligonucleotide containing eight degenerate nucleotides. As the sequencing capacity increased, the fold coverage became more evenly distributed. The small window shows the fold coverage over the range 0–50x for 0.1M and 1M reads. The arrows indicate the coverage ratio for maximum diversity in the current design ($4^8 = 65536$). The discrepancy between the real performance and the simulation lies in the errors in 454 (for the generation of clonal oligonucleotides) and Illumina sequencing (the decoding process).

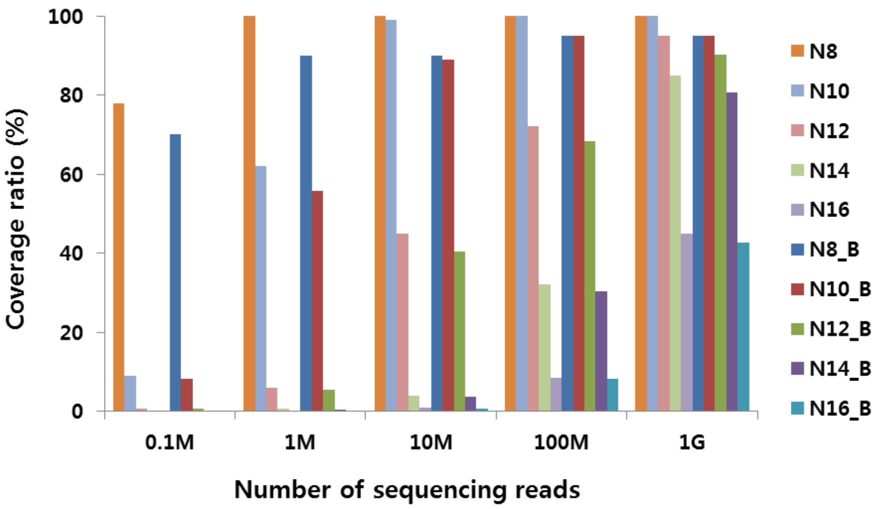

Figure S5. A comparison of the synthetic bias in the coverage ratio simulation. In the case of the unequal probability of obtaining each DNA sequence, the coverage ratio is lower than that of the uniform case. The unequal sampling probability was modelled using an empirical distribution in the current study. N8_B indicates the case with 8 bp degenerate oligonucleotides with a biased distribution of the contents. The error bars represent mean + s.e.m from 10 Monte Carlo simulations.

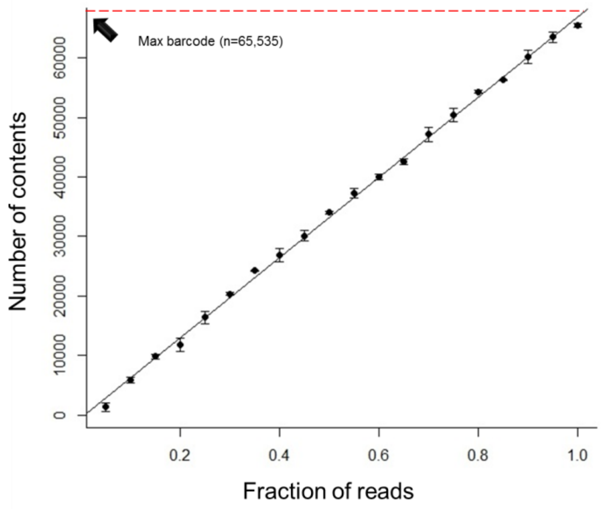

Figure S6. Downsampling of the reads (from the initially sequenced oligonucleotide) to predict the degree of saturation. We utilized a down-sampling strategy to explore whether the saturation of a unique number of degenerate oligonucleotides can be achieved. We then extrapolated the fraction of reads required to gain the maximum number of content (n = 65,536) for degenerate oligonucleotides and found that it was close to saturation using a linear regression (x1.0023 of reads compared with original data were required). The black error bars represent standard deviations of the 10 iterations of random downsampling.

Subsequent code

Previous character

Rule	0	1	2	3
"T"	**C**	**G**	**A**	**T**
"C"	**G**	**A**	**T**	**C**
"G"	**A**	**T**	**C**	**G**
"A"	**T**	**C**	**G**	**A**

Figure S7. Base-4 system to DNA encoding strategy. To encode Huffman-encoded base-4 digits to DNA sequences, we utilized simple rule to minimize repetitive sequences. Set “T”, “C”, “G” and “A” to 0, 1, 2 and 3 respectively. Then, subsequent base was converted as the rule on the above table. For example, “01” could be converted to “TG”.
